# Factors Associated With Quality of Life Among Older Prostate Cancer Survivors: NIH All of Us Research Program

**DOI:** 10.1093/geroni/igaf122.845

**Published:** 2025-12-31

**Authors:** Tung Sung Tseng, Masuma Mannan, Yu-Wen Chiu, Ya-Hsin Li, Hui-Yi Lin

**Affiliations:** Louisiana State University School of Public Health, New Orleans, Louisiana, United States; Louisiana State University Health Sciences Center School of Public Health, New Orleans, Louisiana, United States; Louisiana State University Health Sciences Center School of Public Health, Mew Orleans, Louisiana, United States; Chung Shan Medical University, Taichung, Taichung, Taiwan (Republic of China); Louisiana State University Health Sciences Center, New Orleans, Louisiana, United States

## Abstract

Prostate cancer (PCa) is the first leading cause of cancer incidence and the 2nd leading cause of cancer deaths among American men in 2024. Most of the PCa patients have long-term survivorship, and the impact of the disease and quality of life (QoL) becomes a crucial aspect of well-being and overall survival. This study included 6,426 PCa patients, mean age 75 (SD = 8.4), from the NIH All of Us research program. Results showed that 558 (8.8%) PCa patients reported low QoL, 1,324 (20.9%) reported poor physical health and 534 (9.0%) reported poor mental health. The multivariable models indicate that African Americans, those not married or living with a partner, those with lower education levels, low income, current smokers, high fatigue levels, and poor social role reported lower QoL. Age (OR = 0.98, p < 0.0001), overweight (OR = 0.82 vs. under or normal weight, p < 0.0001), smoking status (OR = 1.34, p < 0.040 for current smokers), alcohol consumption (OR = 0.59, p < 0.0001 for high consumption), social function (OR = 6.64 for poor function, p < 0.0001), and fatigue (OR = 4.62 for high level, p < 0.0001) significantly impact on poor physical health. This study identified several factors (especially social function and fatigue level) that had a major impact on QoL and related health measures for PCa patients. These findings may help healthcare providers better understand PCa patients’ overall well-being and help physicians and PCa patients make medical decisions accordingly. This consideration can improve PCa patient satisfaction and outcomes.

